# Prostatic Acid Phosphatase Is Expressed in Peptidergic and Nonpeptidergic Nociceptive Neurons of Mice and Rats

**DOI:** 10.1371/journal.pone.0008674

**Published:** 2010-01-13

**Authors:** Bonnie Taylor-Blake, Mark J. Zylka

**Affiliations:** Department of Cell and Molecular Physiology, UNC Neuroscience Center, University of North Carolina, Chapel Hill, North Carolina, United States of America; Emory University, United States of America

## Abstract

Thiamine monophosphatase (TMPase, also known as Fluoride-resistant acid phosphatase or FRAP) is a classic histochemical marker of small- to medium-diameter dorsal root ganglia (DRG) neurons and has primarily been studied in the rat. Previously, we found that TMPase was molecularly identical to Prostatic acid phosphatase (PAP) using mice. In addition, PAP was expressed in a majority of nonpeptidergic, isolectin B4-binding (IB4^+^) nociceptive neurons and a subset of peptidergic, calcitonin gene-related peptide-containing (CGRP^+^) nociceptive neurons. At the time, we were unable to determine if PAP was present in rat DRG neurons because the antibody we used did not cross-react with PAP in rat tissues. In our present study, we generated a chicken polyclonal antibody against the secretory isoform of mouse PAP. This antibody detects mouse, rat and human PAP protein on western blots. Additionally, this antibody detects PAP in mouse and rat small- to medium-diameter DRG neurons and axon terminals in lamina II of spinal cord. In the rat, 92.5% of all PAP^+^ cells bind the nonpeptidergic marker IB4 and 31.8% of all PAP^+^ cells contain the peptidergic marker CGRP. Although PAP is found in peptidergic and nonpeptidergic neurons of mice and rats, the percentage of PAP^+^ neurons that express these markers differs between species. Moreover, PAP^+^ axon terminals in the rat partially overlap with Protein kinase Cγ (PKCγ^+^) interneurons in dorsal spinal cord whereas PAP^+^ axon terminals in the mouse terminate dorsal to PKCγ^+^ interneurons. Collectively, our studies highlight similarities and differences in PAP localization within nociceptive neurons of mice and rats.

## Introduction

It has long been known that small- to medium-diameter DRG neurons contain an acid phosphatase called Thiamine monophosphatase (TMPase; also known as Fluoride-resistant acid phosphatase or FRAP) [1], [2], [3], [4], [5], [6]. TMPase activity was visualized histochemically by incubating tissue sections with phosphorylated substrates then detecting the deposition of an insoluble lead precipitate on cell bodies and axons. TMPase was arguably the first marker for nociceptive DRG neurons and was extensively studied through the 1980s, primarily in rat tissues. TMPase was also found in small- and medium-diameter DRG neurons of other mammalian species, including mouse, rabbit, cat, dog, monkey, cow and human, suggesting a species-conserved function for this enzyme [3], [4].

In general, nociceptive (“pain-sensing”) neurons can be divided into peptidergic and nonpeptidergic subsets that differ molecularly, anatomically, developmentally and functionally [7], [8], [9], [10], [11], [12]. TMPase was originally thought to be a marker of nonpeptidergic DRG neurons based on limited (1%) to no overlap with the peptidergic marker Substance P [2], [13]. However, subsequent studies in the rat revealed that TMPase was found in most nonpeptidergic neurons and a subset of peptidergic neurons. Specifically, TMPase was extensively co-localized in cells and axon terminals with the nonpeptidergic neuron marker IB4 and partially co-localized (30–50%) with CGRP, a more broadly expressed marker of peptidergic DRG neurons [14], [15], [16]. At the time, it was not known what gene encoded TMPase so experiments examining overlap could not be performed using double-label immunofluorescence.

Recently, we found that TMPase was molecularly identical to the transmembrane isoform of Prostatic acid phosphatase (PAP; also known as ACPP). PAP was expressed in a majority of all nonpeptidergic neurons and a subset of peptidergic nociceptive neurons in the mouse [17]. Moreover, we found that PAP functioned as an ectonucleotidase with relative specificity adenosine 5′-monophosphate [17], [18].

There are two isoforms of PAP, a secreted isoform and a transmembrane isoform [17], [19], [20]. Both isoforms are identical at the amino acid level, including the N-terminal signal peptide and catalytic region, but differ at the C-terminus because of alternative splicing. As part of our previous study, we used antibodies generated against the secretory isoform of human PAP to detect PAP in mouse DRG neurons and axon terminals in spinal cord [17]. Our studies with PAP/TMPase in mice, combined with previous studies on TMPase in rats, suggested PAP should be present in rat DRG neurons. However, we were unable to detect PAP immunoreactivity in sections from rat DRG or spinal cord (unpublished observations). Two other groups were similarly unable to detect PAP-like immunoreactivity in rat DRG using antibodies directed against human PAP [2], [3]. Here, we generated a new antibody that recognizes PAP in mouse and rat tissues and used it for comparative studies in DRG and dorsal spinal cord.

## Results and Discussion

### Generation and Validation of Chicken Antibodies to Mouse PAP Protein

PAP was discovered over 70 years ago and was used as a diagnostic marker for prostate cancer in humans [21], [22], [23], [24], [25], [26]. Over time, numerous antibodies were raised against human PAP. Human (h)PAP is >80% identical to mouse (m) and rat (r) PAP (Table 1) [20]. This similarity suggested that some of the antibodies raised against hPAP might detect PAP in rodent tissues. We thus tested four commercially available antibodies directed against hPAP (including Biømeda-V2005, Sigma-P5664, Abcam-ab9381, Axcell-YIA7411; we could not find commercially available antibodies directed against mouse or rat PAP). Of these, only the Biømeda antibody detected PAP in mouse DRG neurons and axon terminals [17]. However, this antibody did not recognize PAP in rat DRG neurons or spinal axon terminals (data not shown). This lack of immunoreactivity could be due to the lower percent identity/similarity between hPAP and rPAP relative to hPAP and mPAP (Table 1). PAP is a glycosylated protein [23], so differences in glycosylation might also affect immunoreactivity. While our research with PAP was in progress [17], Biømeda went out of business, making it impossible to obtain this antibody for further studies.

**Table 1:** Amino acid identity and similarity between the secretory isoforms of mouse, rat and human PAP.

		% Identity		
		mPAP	rPAP	hPAP
	mPAP	-----	88	83
% Similarity	rPAP	94	-----	81
	hPAP	91	89	-----

Calculated using BLASTP with GenBank accession #'s NP_062781.2 (mPAP), NP_064457.1 (rPAP) and NP_001090.2 (hPAP). The less conserved N- and C-terminal regions were not included in these alignments, resulting in higher percent identity values relative to a previous study with rPAP and hPAP [20].

**Figure 1:**
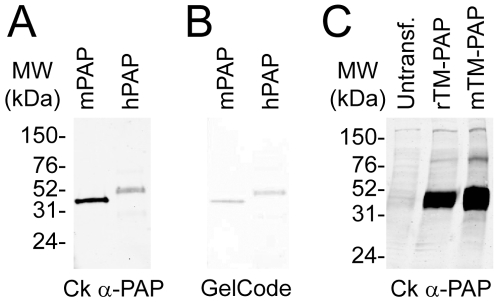
Chicken antibody detects mouse, rat and human PAP on western blots. (A) Western blot containing purified recombinant mPAP protein and pure hPAP protein probed with chicken (Ck) anti-PAP antibody. (B) Duplicate gel stained with GelCode blue to confirm that equivalent amounts of protein were loaded. (C) Western blot of cell lysates from untransfected HEK 293 cells and HEK 293 cells transfected with rTM-PAP or mTM-PAP.

To generate a polyclonal antibody that reliably detects PAP in mouse tissues, we immunized chickens with the secretory isoform of full-length recombinant mPAP protein, purified as described previously [18]. This resulted in a high-titer antibody that recognized secretory mPAP protein in enzyme-linked immunosorbent assays (data not shown) and on western blots (Figure 1A). This antibody also recognized secretory hPAP protein on western blots, although the signal intensity was lower (Figure 1A). Since similar amounts of mPAP and hPAP protein were loaded, confirmed by staining a duplicate gel for total protein (Figure 1B), this suggested our chicken antibody had greater specificity for mPAP over hPAP protein. Lastly, this antibody recognized the transmembrane isoform of mouse PAP (mTM-PAP) and rat PAP (rTM-PAP) on western blots (Figure 1C).

**Figure 2:**
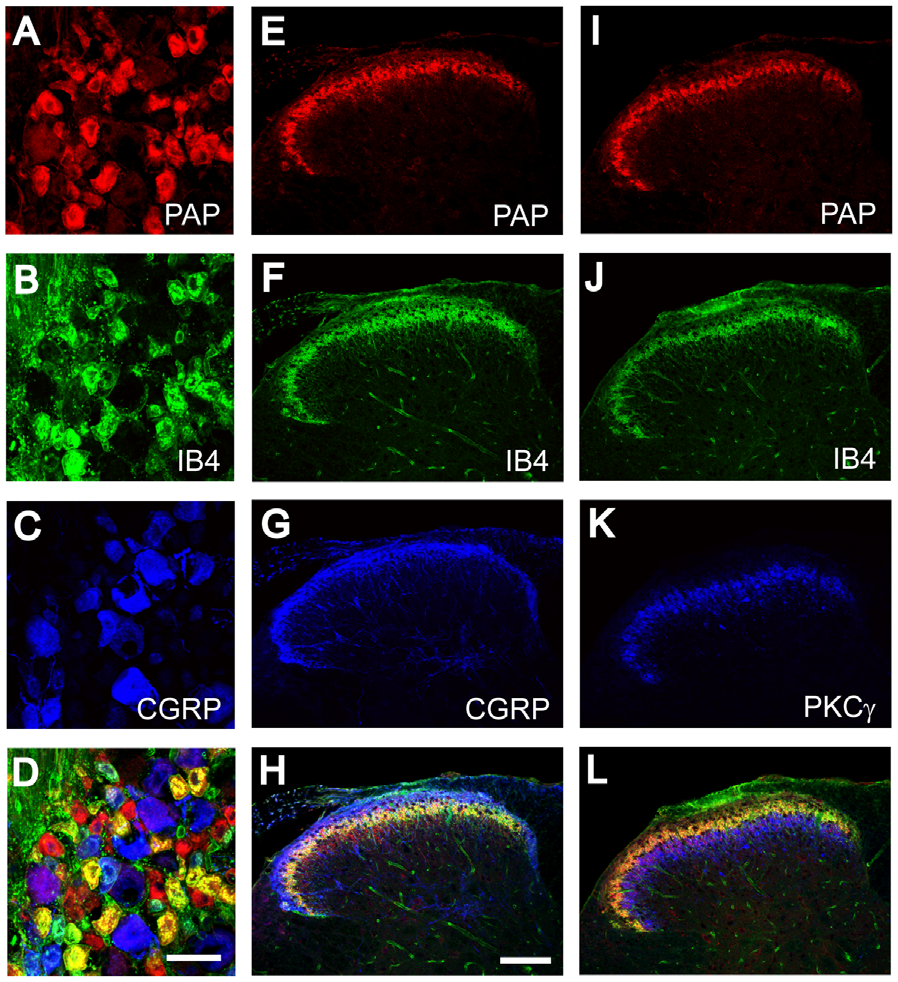
Chicken antibody detects PAP in mouse DRG neurons and dorsal spinal cord. (A–D) Sections from mouse L4-L6 DRG and (E–L) lumbar spinal cord were stained with chicken anti-PAP antibodies (red) and with antibodies against various sensory neuron markers and spinal interneuron marker PKCγ (blue, green). (D, H, L) Merged images. All images were acquired by confocal microscopy. Scale bar in (D) 50 µm, (H) 100 µm.

We previously found that PAP was co-localized in a majority of all IB4^+^ nonpeptidergic neurons and a subset of all CGRP^+^ peptidergic neurons in the mouse using the Biømeda antibody [17]. In addition, we found that PAP was extensively co-localized with IB4 in axon terminals, located in lamina II of dorsal spinal cord. To determine if our chicken antibody recognized PAP in a similar population of DRG neurons and axon terminals, we triple-immunostained mouse DRG and spinal cord with our chicken antibody and various markers (Figure 2). In mouse lumbar DRG, 66.6% of all PAP^+^ cells bound the nonpeptidergic marker IB4 whereas 9.6% of PAP^+^ cells contained the peptidergic marker CGRP^+^ (n = 1289 cells counted) (Figure 2A–D). Conversely, 83.7% of all IB4^+^ cells were PAP^+^ and 14.8% of CGRP^+^ cells were PAP^+^ (n = 1289 cells counted). In the mouse dorsal spinal cord, PAP immunostaining overlapped extensively with IB4 and was concentrated in axon terminals within lamina II (Figure 2E, F, H). In contrast, there was limited overlap between PAP and CGRP in lamina II (Figure 2E, G, H). PKCγ marks a class of interneurons in lamina II_inner_ and lamina III that are implicated in neuropathic pain mechanisms and the detection of innocuous stimuli [27], [28], [29], [30]. PAP axonal staining was dorsal to and largely non-overlapping with the band of PKCγ interneurons neurons (Figure 2I, K, L). As controls for the experiments described above, no immunostaining was observed in DRG neurons or spinal cord when using preimmune serum or when the chicken anti-PAP antibody was omitted (data not shown). Taken together, the cellular and axonal distribution of PAP, as revealed with our chicken antibody, was similar to our previous study with the Biømeda antibody [17]. These results indicated that our chicken antibody reliably detects PAP in mouse tissues.

### PAP Marks Peptidergic and Nonpeptidergic Nociceptive Neurons in the Rat

**Figure 3:**
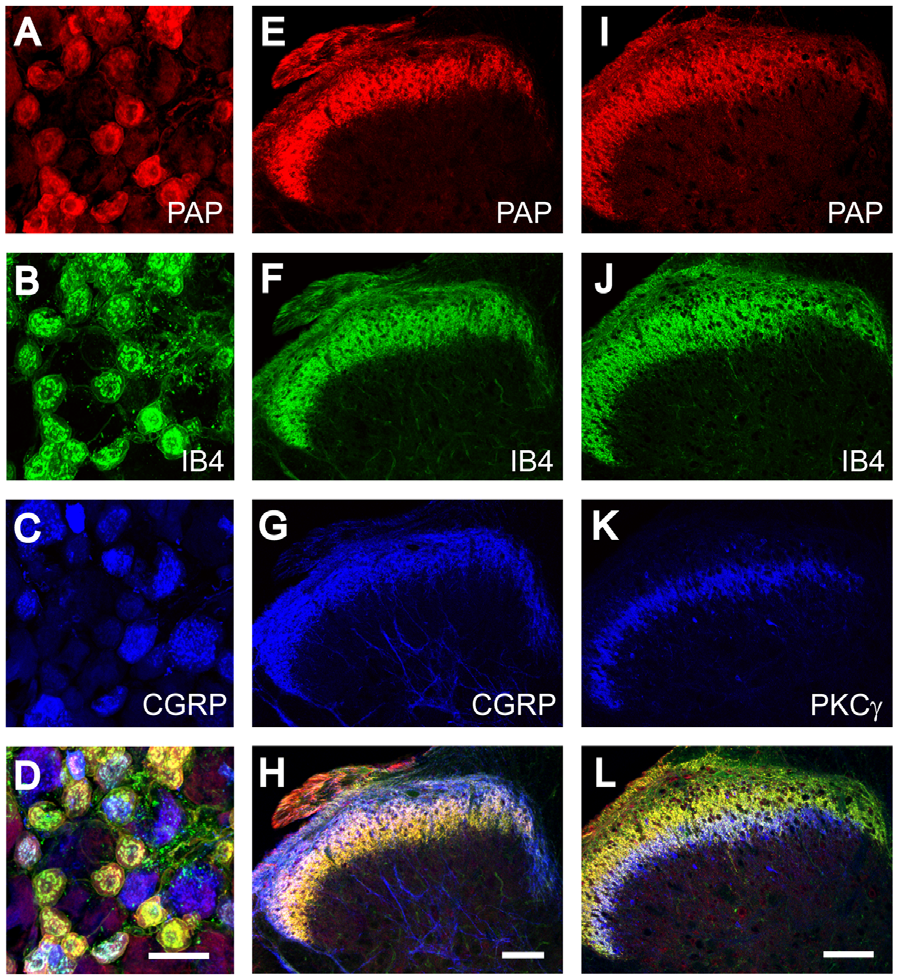
Chicken antibody detects PAP in rat DRG neurons and dorsal spinal cord. (A–D) Sections from rat L4-L6 DRG and (E–L) lumbar spinal cord were stained with chicken anti-PAP antibodies (red) and with antibodies against various sensory neuron markers and spinal interneuron marker PKCγ (blue, green). (D, H, L) Merged images. All images were acquired by confocal microscopy. Scale bar in (D) 50 µm, (H) 100 µm.

We next triple-immunostained lumbar DRG and spinal cord sections from the rat to determine if our polyclonal antibody also recognized PAP in rat tissues. The chicken anti-PAP antibody labeled a subset of small- to medium-diameter neurons in rat DRG and labeled axon terminals in lamina II of the spinal cord (Figure 3A, E, I). In lumbar DRG, 92.5% of the PAP^+^ cells bound the nonpeptidergic marker IB4^+^ whereas 31.8% of PAP^+^ cells contained the peptidergic marker CGRP^+^ (n = 443 cells counted per condition) (Figure 3A–D). Conversely, 80.2% of all IB4^+^ were PAP^+^ and 41.5% of all CGRP^+^ neurons were PAP^+^ (n = 356 cells counted). These percentages obtained by triple immunofluorescence labeling closely matched previous studies where TMPase (now known to be PAP) was co-localized with markers in sections from the rat. Specifically, 95% of all TMPase^+^ cells were IB4^+^ and 50% of all TMPase^+^ cells were CGRP^+^ in the rat [14], [15], [31].

In the dorsal spinal cord, there was extensive co-localization between PAP^+^ and IB4^+^ axon terminals in lamina II and partial overlap with CGRP^+^ axon terminals (Figure 3E–H). Unlike the mouse where PAP^+^ terminals and PKCγ were largely non-overlapping, there was significant overlap between PAP^+^ axon terminals and PKCγ interneurons in the rat (Figure 3I, K, L). Indeed, we and others previously found that nonpeptidergic (IB4^+^) afferents of rats and mice terminate in different locations relative to PKCγ^+^ interneurons [8], [27]; also see Figures 2I–L and 3I–L.

Taken together, our studies indicate that PAP is found in peptidergic and nonpeptidergic neurons of the rat, although the extent of co-localization (both in DRG neurons and dorsal spinal cord) differs with species. Notably, a larger percentage (31.8%) of PAP^+^ neurons in the rat contains the peptidergic marker CGRP when compared to mouse (14.8%). This correlates with a more extensive overlap between PAP^+^ and CGRP^+^ axon terminals in spinal cord of rat (compare Figure 2E–H with Figure 3E–H). Considering the role of CGRP in inflammatory processes and heat sensitivity [32], [33], [34], a species difference in CGRP expression could differentially affect how rats and mice respond to inflammatory and thermal stimuli. Additionally, we and others found that rats and mice differentially express several other pain-relevant genes in peptidergic and nonpeptidergic nociceptive neurons, including the noxious heat receptor TRPV1 and Mas-related G protein-coupled receptors [35], [36], [37]. These species differences highlight the need for caution when extrapolating physiological and behavioral findings that pertain to nociception from one species to the other.

## Materials and Methods

### Chicken Anti-PAP Antibody Production

Recombinant mPAP protein containing a C-terminal thrombin-hexahistidine epitope tag was purified using the baculovirus expression system as previously described [18] and used to immunize hens (Aves Labs). The IgY fraction was purified and used in this study. Our chicken anti-PAP antibody is commercially available through Aves Labs (Cat #PAP).

### Western Blots

Recombinant mPAP protein and hPAP protein (Millipore/Chemicon Cat. #AG60) were separated by SDS-PAGE (0.2–1.0 µg protein/lane). HEK 293 cells were transfected as described [38] with pcDNA3.1 expression constructs containing full-length rTM-PAP [19] or mTM-PAP [17]. Cell lysates were prepared 48 h post transfection in RIPA buffer containing protease inhibitors (Roche Complete Mini, Cat. #1836153) and separated by SDS-PAGE. Samples were not boiled prior to loading. Gels were stained for total protein with GelCode Blue (Pierce/Thermo Scientific, Cat. #24590) or were western blotted onto nitrocellulose. Blots were blocked with 2% cold water fish gelatin, then probed with chicken anti-PAP (1∶40,000) overnight at 4°C, washed in TBS-T (0.1 M Tris, pH 7.5, 0.16 M NaCl, 0.1% Tween-20), then incubated with 1∶5000 goat anti-chicken IgY IRDye 800 (Rockland, Cat #603-132-126) for 1 hr. Blots were imaged on a Li-cor Odyssey system.

### Immunofluorescence

All procedures involving vertebrate animals were approved by the Institutional Animal Care and Use Committee at the University of North Carolina at Chapel Hill.

Adult male mice (C57BL/6, 6–8 weeks) were sacrificed by decapitation. Adult male Sprague-Dawley rats were sacrificed by overdosing with pentobarbital. Lumbar DRG and spinal cord were removed and immersed in 4% paraformaldehyde in 0.1 M phosphate buffer (pH 7.4) for 4 h (mouse DRG), 8 h (mouse spinal cord) or 12 h (rat tissues). Tissues were cryoprotected in 30% sucrose in 0.1 M phosphate buffer after immersion-fixation. DRG were sectioned frozen at 20 µm, collected on Superfrost plus slides then immunostained on slides. Spinal cords were sectioned at 30 µm and processed free-floating. Sections were treated with 1% hydrogen peroxide in phosphate-buffered saline (pH 7.4) for 30 min to reduce endogenous peroxidase. A high-salt (2.7%) Tris-buffered saline containing 0.3% Triton-X (TBS/TX) was used for all subsequent steps. Sections were incubated overnight at 4°C with primary antibodies diluted in 10% normal donkey serum in TBS/TX. Primary antibodies included: chicken anti-PAP (1∶4,000), rabbit anti-CGRP (1∶750; Bachem/Peninsula, T-4032) and rabbit anti-PKCγ (1∶750; Santa Cruz, C-19, sc-211). Chicken anti-PAP staining was revealed through the use of a biotinylated secondary (Jackson ImmunoResearch; 703-065-155), ABC complex (Standard Elite, Vector Laboratories, PK-6100) and TSA-Cy3 amplification (PerkinElmer, SAT704A). CGRP and PKCγ staining were revealed through the use of Cy5-coupled secondary antibodies. Sections were treated with IB4-Alexa Fluor-488 (Invitrogen; I21412) after TSA-Cy3 amplification because IB4-staining was difficult to detect if tissues were incubated with IB4 before TSA-Cy3 amplification. Images were obtained using a Zeiss LSM 510 confocal microscope.

## References

[1] Colmant HJ (1959). Aktivitatsschwankungen der sauren Phosphatase im Ruckenmark und den Spinalganglien der Ratte nach Durchschneidung des Nervus ischiadicus.. Arch Psychiat Nervnekr.

[2] Dodd J, Jahr CE, Hamilton PN, Heath MJ, Matthew WD (1983). Cytochemical and physiological properties of sensory and dorsal horn neurons that transmit cutaneous sensation.. Cold Spring Harb Symp Quant Biol.

[3] Silverman JD, Kruger L (1988). Acid phosphatase as a selective marker for a class of small sensory ganglion cells in several mammals: spinal cord distribution, histochemical properties, and relation to fluoride-resistant acid phosphatase (FRAP) of rodents.. Somatosens Res.

[4] Sanyal S, Rustioni A (1974). Phosphatases in the substantia gelatinosa and motoneurones: a comparative histochemical study.. Brain Res.

[5] Knyihar E, Gerebtzoff MA (1970). Localisation ultrastructurale de l' isoenzyme fluororesistant de la phosphatase acide dans la moelle epiniere de rat.. Bull Assoc Anat.

[6] Knyihar-Csillik E, Bezzegh A, Boti S, Csillik B (1986). Thiamine monophosphatase: a genuine marker for transganglionic regulation of primary sensory neurons.. J Histochem Cytochem.

[7] Molliver DC, Wright DE, Leitner ML, Parsadanian AS, Doster K (1997). IB4-binding DRG neurons switch from NGF to GDNF dependence in early postnatal life.. Neuron.

[8] Zylka MJ, Rice FL, Anderson DJ (2005). Topographically distinct epidermal nociceptive circuits revealed by axonal tracers targeted to Mrgprd.. Neuron.

[9] Cavanaugh DJ, Lee H, Lo L, Shields SD, Zylka MJ (2009). Distinct subsets of unmyelinated primary sensory fibers mediate behavioral responses to noxious thermal and mechanical stimuli.. Proc Natl Acad Sci U S A.

[10] Hunt SP, Mantyh PW (2001). The molecular dynamics of pain control.. Nat Rev Neurosci.

[11] Woolf CJ, Ma Q (2007). Nociceptors–noxious stimulus detectors.. Neuron.

[12] Hunt SP, Rossi J (1985). Peptide- and non-peptide-containing unmyelinated primary afferents: the parallel processing of nociceptive information.. Philos Trans R Soc Lond B Biol Sci.

[13] Nagy JI, Hunt SP (1982). Fluoride-resistant acid phosphatase-containing neurones in dorsal root ganglia are separate from those containing substance P or somatostatin.. Neuroscience.

[14] Wang H, Rivero-Melian C, Robertson B, Grant G (1994). Transganglionic transport and binding of the isolectin B4 from Griffonia simplicifolia I in rat primary sensory neurons.. Neuroscience.

[15] Carr PA, Yamamoto T, Nagy JI (1990). Calcitonin gene-related peptide in primary afferent neurons of rat: co-existence with fluoride-resistant acid phosphatase and depletion by neonatal capsaicin.. Neuroscience.

[16] Dalsgaard CJ, Ygge J, Vincent SR, Ohrling M, Dockray GJ (1984). Peripheral projections and neuropeptide coexistence in a subpopulation of fluoride-resistant acid phosphatase reactive spinal primary sensory neurons.. Neurosci Lett.

[17] Zylka MJ, Sowa NA, Taylor-Blake B, Twomey MA, Herrala A (2008). Prostatic acid phosphatase is an ectonucleotidase and suppresses pain by generating adenosine.. Neuron.

[18] Sowa NA, Vadakkan KI, Zylka MJ (2009). Recombinant mouse PAP Has pH-dependent ectonucleotidase activity and acts through A(1)-adenosine receptors to mediate antinociception.. PLoS ONE.

[19] Quintero IB, Araujo CL, Pulkka AE, Wirkkala RS, Herrala AM (2007). Prostatic acid phosphatase is not a prostate specific target.. Cancer Res.

[20] Roiko K, Janne OA, Vihko P (1990). Primary structure of rat secretory acid phosphatase and comparison to other acid phosphatases.. Gene.

[21] Gutman AB, Gutman EB (1938). An “acid” phosphatase occurring in the serum of patients with metastasizing carcinoma of the prostate gland.. J Clin Invest.

[22] Ostrowski WS, Kuciel R (1994). Human prostatic acid phosphatase: selected properties and practical applications.. Clin Chim Acta.

[23] Kaija H, Patrikainen LOT, Alatalo SL, Vaananen HK, Vihko PT, Seibel MJ, Robins SP, Bilezikian JP (2006). Acid Phosphatases.. Dynamics of Bone and Cartilage Metabolism: Academic Press.

[24] Taira A, Merrick G, Wallner K, Dattoli M (2007). Reviving the acid phosphatase test for prostate cancer.. Oncology (Williston Park).

[25] Vihko P, Schroeder FH, Lukkarinen O, Vihko R (1982). Secretion into and elimination from blood circulation of prostate specific acid phosphatase, measured by radioimmunoassay.. J Urol.

[26] Cooper JF, Foti A, Imfeld H (1976). Production of specific antibody to purified prostatic acid phosphatase.. Urol Res.

[27] Neumann S, Braz JM, Skinner K, Llewellyn-Smith IJ, Basbaum AI (2008). Innocuous, not noxious, input activates PKCgamma interneurons of the spinal dorsal horn via myelinated afferent fibers.. J Neurosci.

[28] Malmberg AB, Chen C, Tonegawa S, Basbaum AI (1997). Preserved acute pain and reduced neuropathic pain in mice lacking PKCgamma.. Science.

[29] Polgár E, Fowler JH, McGill MM, Todd AJ (1999). The types of neuron which contain protein kinase C gamma in rat spinal cord.. Brain Res.

[30] Mori M, Kose A, Tsujino T, Tanaka C (1990). Immunocytochemical localization of protein kinase C subspecies in the rat spinal cord: light and electron microscopic study.. J Comp Neurol.

[31] Silverman JD, Kruger L (1990). Selective neuronal glycoconjugate expression in sensory and autonomic ganglia: relation of lectin reactivity to peptide and enzyme markers.. J Neurocytol.

[32] Mogil JS, Miermeister F, Seifert F, Strasburg K, Zimmermann K (2005). Variable sensitivity to noxious heat is mediated by differential expression of the CGRP gene.. Proc Natl Acad Sci U S A.

[33] Gamse R, Saria A (1985). Potentiation of tachykinin-induced plasma protein extravasation by calcitonin gene-related peptide.. Eur J Pharmacol.

[34] Richardson JD, Vasko MR (2002). Cellular mechanisms of neurogenic inflammation.. J Pharmacol Exp Ther.

[35] Zwick M, Davis BM, Woodbury CJ, Burkett JN, Koerber HR (2002). Glial cell line-derived neurotrophic factor is a survival factor for isolectin B4-positive, but not vanilloid receptor 1-positive, neurons in the mouse.. J Neurosci.

[36] Zylka MJ, Dong X, Southwell AL, Anderson DJ (2003). Atypical expansion in mice of the sensory neuron-specific Mrg G protein-coupled receptor family.. Proc Natl Acad Sci U S A.

[37] Caterina MJ, Schumacher MA, Tominaga M, Rosen TA, Levine JD (1997). The capsaicin receptor: a heat-activated ion channel in the pain pathway.. Nature.

[38] Campagnola L, Wang H, Zylka MJ (2008). Fiber-coupled light-emitting diode for localized photostimulation of neurons expressing channelrhodopsin-2.. J Neurosci Methods.

